# Implication of the 2014 World Health Organization Integrated Management of Childhood Illness Pneumonia Guidelines with and without pulse oximetry use in Malawi: A retrospective cohort study

**DOI:** 10.12688/gatesopenres.13963.2

**Published:** 2023-11-08

**Authors:** Shubhada Hooli, Charles Makwenda, Norman Lufesi, Tim Colbourn, Tisungane Mvalo, Eric D. McCollum, Carina King

**Affiliations:** 1 Department of Pediatrics, Division of Emergency Medicine, Baylor College of Medicine, Houston, TX, USA; 2 Parent and Child Health Initiative, Lilongwe, Malawi; 3 Republic of Malawi Ministry of Health, Lilongwe, Malawi; 4 Institute for Global Health, University College London, London, UK; 5 University of North Carolina Project Malawi, Lilongwe, Malawi; 6 Department of Pediatrics, University of North Carolina School of Medicine, Chapel Hill, NC, USA; 7 Department of International Health, Johns Hopkins Bloomberg School of Public Health, Baltimore, MD, USA; 8 Global Program in Respiratory Sciences, Eudowood Division of Pediatric Respiratory Sciences, Department of Pediatrics, School of Medicine, Johns Hopkins University, Baltimore, MD, USA; 9 Department of Global Public Health, Karolinska Institute, Stockholm, Sweden

**Keywords:** infant, child, pneumonia, child mortality, oximetry, Malawi, Africa, World Health Organization

## Abstract

**Background:**

Under-5 pneumonia mortality remains high in low-income countries. In 2014 the World Health Organization (WHO) advised that children with chest indrawing pneumonia, but without danger signs or peripheral oxygen saturation (SpO _2_) < 90% be treated in the community, rather than hospitalized. In Malawi there is limited pulse oximetry availability.

**Methods:**

Secondary analysis of 13,413 under-5 pneumonia cases in Malawi. Pneumonia associated case fatality ratios (CFR) were calculated by disease severity under the assumptions of the 2005 and 2014 WHO Integrated Management of Childhood Illness (IMCI) guidelines, with and without pulse oximetry. We investigated if pulse oximetry readings were missing not at random (MNAR).

**Results:**

The CFR of patients classified as having non-severe pneumonia per the 2014 IMCI guidelines doubled under the assumption that pulse oximetry was not available (1.5% without pulse oximetry vs 0.7% with pulse oximetry, P<0.001). When 2014 IMCI guidelines were applied with pulse oximetry and a SpO _2_ < 90% as the threshold for referral and/or admission, the number of cases meeting hospitalization criteria decreased by 70.3%. Unrecorded pulse oximetry readings were MNAR with an adjusted odds for mortality of 4.9 (3.8, 6.3), similar to that of a SpO _2_ < 90%. Although fewer girls were hospitalized, female sex was an independent mortality risk factor.

**Conclusions:**

In Malawi, implementation of the 2014 WHO IMCI pneumonia guidelines, without pulse oximetry, will miss high risk cases. Alternatively, implementation of pulse oximetry may result in a large reduction in hospitalization rates without significantly increasing non-severe pneumonia associated CFR if the inability to obtain a pulse oximetry reading is considered a WHO danger sign.

## Background

Annually over 800,000 deaths of children under 5 years old are attributable to acute lower respiratory infections worldwide ^1^. Due to its high prevalence, pneumonia is one of the primary diseases addressed by the WHO Integrated Management of Childhood Illness (IMCI) guidelines, the standard of care at many frontline, outpatient healthcare facilities in low-income and middle-income countries (LMICs). These guidelines rely on health worker recognition of clinical ‘danger signs’ such as inability to feed, lethargy, intractable vomiting, or convulsions to determine if a child should be referred for further evaluation by a trained medical provider at a hospital and receive more intensive treatment. Two key pneumonia case classification policy changes were made in the 2014 IMCI revision. First, when pulse oximetry is available, children aged 2–59 months old with a peripheral oxyhemoglobin saturation (SpO _2_) <90% (i.e., hypoxemia) should be referred for hospital care. Second, children with chest indrawing, but no general danger signs, were reclassified as appropriate for outpatient management with oral antibiotics, even when SpO _2_ is not measured.

There have been several attempts to determine if chest indrawing pneumonia, without the presence of general danger signs, can be appropriately managed as an outpatient ^2– 6^. One effort by Agweyu *et al.* retrospectively examined the implication of shifting hospitalized Kenyan children with chest indrawing but without general danger signs into outpatient care. The authors reported that children previously categorized as severe pneumonia (i.e., chest-indrawing but no general danger signs) had an in-hospital case fatality ratio (CFR) of 2.7% ^2^. Similar to many LMIC healthcare settings, pulse oximetry was unavailable, so the authors could not comment on the impact of identifying hypoxemia.

The Malawi Ministry of Health (MOH) adopted the 2014 IMCI pneumonia guidelines in 2018. However, based on anecdotal evidence [SH], some clinicians are hesitant to adopt the revised guidelines into practice due to concerns that the change may result in increased out of hospital complications and/or community deaths. This may in part stem from pulse oximetry’s limited availability in outpatient settings throughout Malawi, and a high proportion of children dying from pneumonia at home after seeking hospital care ^7, 8^. A non-inferiority trial conducted in Malawi showed HIV-uninfected children with chest indrawing pneumonia could be safely treated as outpatients with 3 versus 5 days of amoxicillin. However, all enrolled children were screened with pulse oximetry and none had a SpO _2_<90% ^4^. In a study linking community health worker (CHW) and outpatient pneumonia episodes with 30-day mortality, augmentation of existing health centre guidelines with pulse oximetry identified high risk cases that would otherwise be missed by clinical guidelines alone ^8^. The current CHW guidelines, unlike those for outpatient settings, recommend hospital referral of children with chest indrawing. The added value of pulse oximetry for identifying referral eligible children was less when chest indrawing triggered referral ^9^.

To some degree chest indrawing may be a clinical marker of respiratory insufficiency or impending failure. Chest indrawing is the paradoxical inward pulling of the soft tissue below the ribs during inspiration. It often occurs due to increasing negative intrapleural pressure generated by patients attempting to expand stiff, diseased lungs, although in younger children and infants with more compliant, cartilaginous chest walls it may also occur when the lungs are healthy. Chest indrawing increases lung tidal volumes and minute ventilation to improve overall ventilation ^10^. Aside from respiratory rate and SpO _2_, chest indrawing is the only indicator of lung mediated respiratory distress in the 2005 IMCI guidelines. The 2014 IMCI guidelines do not contain any lower airway sign that triggers hospital referral in settings where pulse oximetry is not used.

We aimed to determine the impact of implementing the 2005 or 2014 IMCI pneumonia guidelines on inpatient case fatality rates with either universal or no pulse oximetry availability in routine care settings.

## Methods

### Setting

Malawi is a low-income country in southeastern sub-Saharan Africa with a high prevalence of malaria and HIV. Study sites were in the central region, in Mchinji and Lilongwe districts. Data was collected from four rural, non-governmental Christian Health Association of Malawi (CHAM) hospitals, two government districts and one regional tertiary referral hospital. At the time of data collection this represented a catchment of 1.3 million people.

### Study design

We conducted a retrospective secondary analysis of hospital inpatient data that was collected prospectively as part of active surveillance, in a real world setting, during a pneumococcal vaccine effectiveness study, between 2012–2014 ^11^. Data collection was supervised and remediation provided intermittently throughout the study period in order to minimize bias. Hospitalization practices reflect Malawi MOH recommendations prior to the 2014 WHO guideline revision of the care of children aged 2–59 months. In 2019 there was a revision of the guidance as it pertains to infants under 2 months old. Children aged 2–59 months routinely diagnosed with pneumonia and hospitalized were included. As HIV status was not consistently recorded amongst all cases, we excluded known HIV-infected and exposed children. Notably, the 2014 IMCI guidelines still recommends hospital referral for HIV-infected and exposed children with chest indrawing. Additionally, we excluded children who were receiving supplemental oxygen at the time of initial SpO _2_ measurement. The study sample size was determined using convenience of access to retrospective data.

### Data collection

Methodology, training and quality assurance methods have been previously described ^11^. Briefly, in 2001 the Malawi MOH implemented a pneumonia surveillance program called the Child Lung Health Program (CLHP) ^12^. As part of CLHP clinicians were supervised by MOH officials including medical officers and the District Medical Officer who was on premises during traditional work hours. Consistent with other district hospitals in Malawi nighttime care was provided by a skeleton staff. At study sites we augmented this existing surveillance program infrastructure by implementing active surveillance with pulse oximetry measurement and data quality assurance. All facilities in the study were visited quarterly by a clinician mentor. Taking a trainer of trainers type of approach, during visits, they would observe pulse oximetry measurements and provide immediate remedial training if any issues were noted.

The Lifebox Foundation (London, UK) supplied medical grade pulse oximeters that have undergone hypoxemia lab testing with adult clip probes (Acare Technology, Xinzhuang, Taiwan, China) which MOH health workers were trained to use via a standardized protocol. These devices are commonly used in surgical care in LMICs. Staff were instructed to place the clip probe on the big toe in children under 10 kg or, in children over 10 kg, a finger. This methodology has been shown to be a reliable and accurate way to measure SpO _2_^13^. Weekly death audits occurred at each study site to ensure that all in-hospital deaths were captured. Study sites were closely supervised, and sites or clinicians with subpar performance received remediation training and supervision. Data was collected using the existing Malawi MOH CLHP case report forms that was subsequently entered into a study-built database, which was routinely checked as part of quality assurance. The original CLHP forms were referred to during data cleaning.

### Analysis

Demographic and clinical characteristics were described as frequencies and proportions. We calculated CFRs with 95% confidence intervals. We compared CFRs by pneumonia classifications based on the 2005 and 2014 WHO guidelines ( Box 1) under two assumptions: without and with pulse oximetry (SpO _2_ <90% considered as a danger sign), using chi-squared tests. If a case had any missing data for the parameters used to classify pneumonia, it was designated as “unable to categorize”. In our previous work analyzing mortality risk factors of Malawian under 2-month olds with acute lower respiratory infections using data from the same study, we found that SpO _2_ was missing not at random (MNAR) and independently associated with in-hospital mortality ^14^. Using logistic regression with multiple imputation with chained equations and pattern mixture modeling we explored if SpO _2_ was MNAR in this cohort of older children as well ^15, 16^. We used *Stata Statistical Software: Release 14*. College Station, TX: StataCorp LP. to conduct all analyses, an open-access alternative that can perform an equivalent function is RStudio.

**Box 1. 2005 vs 2014 WHO IMCI pneumonia hospital referral criteria for 2–59 month olds**

**Table:** 

Pneumonia Classification	2005	2014
Non-severe (outpatient)	Fast breathing ^a^	Fast breathing ^a^ and/or chest indrawing
Severe (hospitalize)	Chest indrawing	Any danger signs ^b^ or SpO _2_<90% ^c^
Very severe (hospitalize)	Any danger signs ^b^	

^a^Fast breathing for age: respiration rate (RR) > 50 breaths per minute (bpm) in 2–11 month olds and RR > 40 bpm in 12–59 month olds; ^b^General danger signs include: unable to drink/feed, convulsions, sleepy/lethargic, vomiting everything, stridor, severe acute malnutrition; ^c^Optional criteria in settings where pulse oximetry is available.

### Ethical considerations

Study ethical approval was provided by the National Health Sciences Research Committee of Malawi (reference: 941, November 14, 2011) and the Ethics Committee of University College London (reference: 2006/002, November 1, 2011). Neither committee required informed consent as the study collected routine clinical data.

## Results

**Figure 1.**
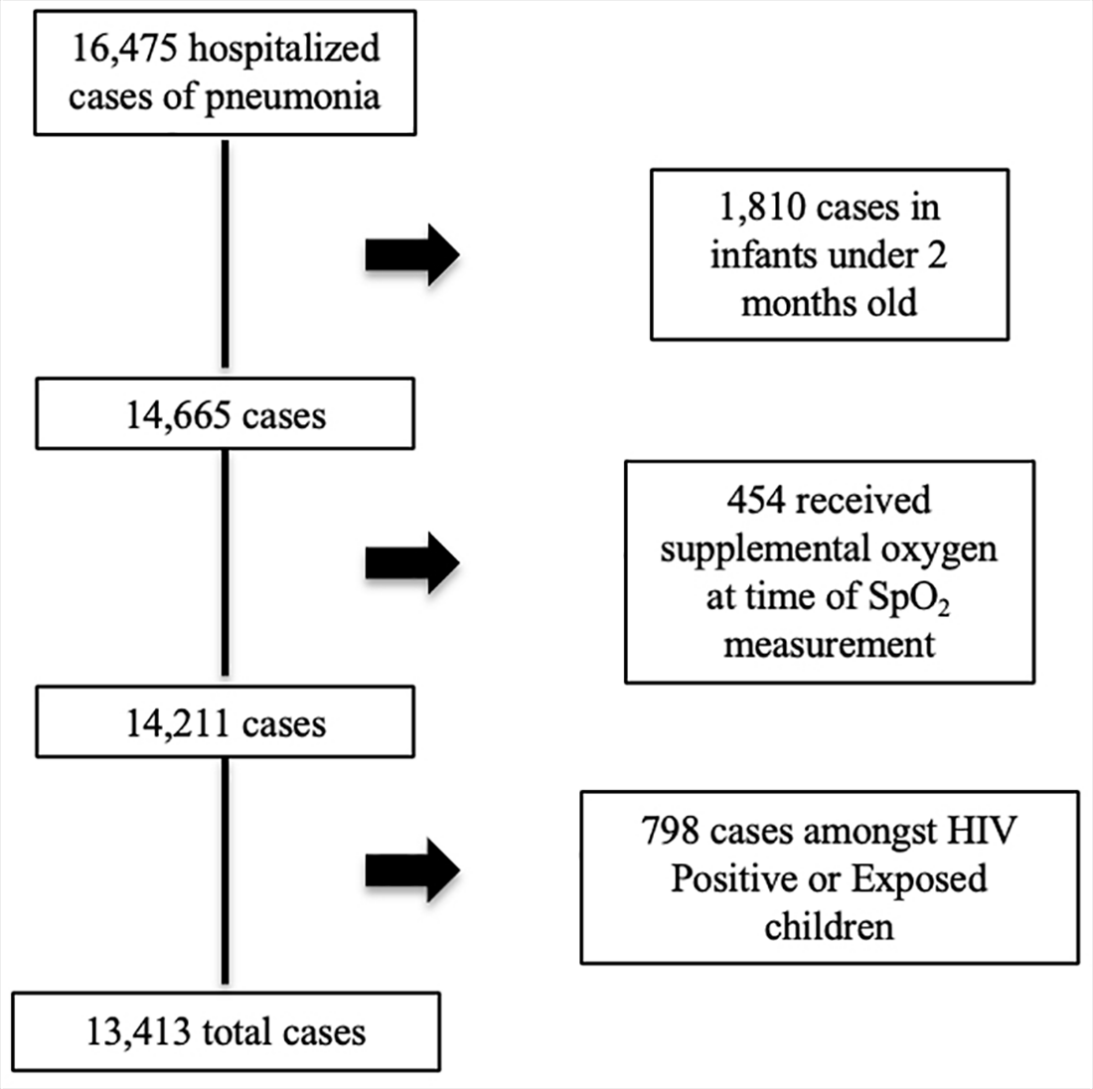
Cases included in analysis.

This analysis includes 13,413 cases with an overall CFR of 2.8% ( Figure 1). Clinical characteristics of the cohort are described in Table 1. A smaller proportion of the hospitalized cases were girls compared with boys (P<0.001), with 3.7% without a recorded sex.

**Table 1.** Demographic and clinical characteristics of children diagnosed with pneumonia.

Clinical Characteristics	N = 13,413
Age, median, months, (IQR)	11 (6-20)
SpO _2_, median, % (IQR)	96 (93-98)
SpO _2_ ≥ 90%, n (%) SpO _2_ < 90%, n (%) Unrecorded SpO _2_, n (%)	10,003 (74.6) 1,249 (9.3) 2,161 (16.1)
Respiratory rate, median (IQR)	58 (50-64)
Weight in kg, mean (SD)	8.9 (2.8)
Male, n (%) Female, n (%)	7,215 (53.8) 5,700 (42.5)
WAZ > -2, n (%) -3 ≤ WAZ < -2, n (%) WAZ ≤ -3, n (%)	10,393 (77.5) 1,224 (9.1) 762 (5.7)
Chest wall indrawing, n (%)	11,185 (83.4)
WHO danger signs *, n (%)	2,230 (16.6)

SpO _2_: peripheral capillary oxyhemoglobin saturation, IQR: interquartile range, CI: confidence interval, SD: standard deviation, WAZ: WHO weight-for-age z-score. *Danger signs include central cyanosis, stridor in a calm child, apnea, or a general danger sign (inability to drink and/or breastfeed, lethargy or unconscious, convulsions)

In Table 2 we compared, using the 2005 and 2014 guidelines, the proportion of cases diagnosed with pneumonia meeting hospital referral and/or admission criteria versus outpatient management and their respective CFRs. The CFRs of children meeting outpatient care criteria per the 2005 and 2014 guidelines were not statistically different (0.8 vs 0.7%). Due to missing data for parameters such as respiratory rate, chest indrawing and danger signs (independent of pulse oximetry reading), 720 cases (5.4%) and 16 deaths (4.3%) with an overall CFR of 2.2% could not be classified as having a specific pneumonia severity. Applying the 2005 guidelines without pulse oximetry, 11,484 cases and 99.1% (347/357) of the pneumonia severity classifiable deaths met hospital referral criteria. Notably, an additional 1,089 cases were unable to be categorized by the 2014 guidelines due to unrecorded pulse oximetry data (deaths=54, CFR 5.0%). In comparison, when using the 2014 guidelines with pulse oximetry, 3,106 cases and 79.2% (240/303) of categorizable deaths met referral criteria. This is a 73.0% reduction in hospital referral cases, however the number of deaths categorized as non-severe increased from 10 (CFR 0.7%) to 63 (CFR 0.8%). When SpO _2_ is treated as an optional criteria, consistent with how the 2014 guidelines are written, the majority of these cases were re-classified as non-severe, outpatient appropriate (N=1,378, deaths = 60, CFR 4.4%). Of these cases 96.9% had chest indrawing (N = 1,335, deaths =59, CFR 4.4%).

**Table 2.** Under 5 Pneumonia In-hospital Case Fatality Ratio Categorized by Pneumonia Severity and Pulse Oximetry Availability.

Pneumonia Classification (N=13,413 Deaths= 373)	2005 WHO IMCI				2014 WHO IMCI			
	No SpO _2_^a^		SpO _2_^b^		SpO _2_^c^		No SpO _2_^d^	
	N (%)	Deaths (CFR)	N (%)	Deaths (CFR)	N (%)	Deaths (CFR)	N (%)	Deaths (CFR)
**Non-severe**	1,209 (9.0%)	10 (0.8%)	1,368 (10.2%)	7 (0.5%)	8,498 (63.4%)	63 (0.7%)	10,463 (78.0%)	155 (1.5%)
**Severe**	9,254 (69.0%)	145 (1.6%)	7,130 (53.2%)	56 (0.8%)	3,106 (23.2%)	240 (7.7%)	2,230 (16.6%)	202 (9.1%)
**Very severe**	2,230 (16.6%)	202 (9.1%)	3,106 (23.2%)	240 (7.7%)	N/A	N/A	N/A	N/A
**Hospital Referral Eligible** **Cases**	11,484 (85.6%)	347 (3.0%)	10,236 (76.3%)	296 (2.9%)	3,106 (23.2%)	240 (7.7%)	2,230 (16.6%)	202 (9.1%)
**Unable to Categorize**	720 (5.4%)	16 (2.2%)	1,809 (13.5%)	70 (3.9%)	1,809 (13.5%)	70 (3.9%)	720 (5.4%)	16 (2.2%)

N = number, % = percentage of total cases, CFR = case fatality ratio; ^a^Guidelines as published, ^b^2005 WHO IMCI with SpO _2 _<90% included as a danger sign and excluding cases with ‘missing’ pulse oximetry, ^c^2014 WHO IMCI guidelines and excluding cases with ‘missing’ pulse oximetry, ^d^2014 WHO IMCI without pulse oximetry

Pulse oximetry is not universally available in outpatient clinics or hospitals in Malawi. Under this assumption we calculated CFRs for differing pneumonia severities without considering SpO _2_. The CFR for patients with 2014 non-severe pneumonia, without pulse oximetry, was twice that of those categorized as having 2005 IMCI guideline non-severe pneumonia (1.5% vs 0.8%, P=0.052). Under the scenario of implementing the 2014 WHO guidelines with pulse oximetry, in comparison to its implementation without pulse oximetry, the CFR of non-severe pneumonia decreased by over 50% (1.5% without pulse oximetry vs 0.7% with pulse oximetry, P<0.001). However it is crucial to emphasize that many of the pre-2014 WHO guideline non-severe pneumonia cases and associated deaths were unable to be categorized due to missing pulse oximetry data. We were unable to categorize the pneumonia severity of 14.5% (N= 54) of the total deaths due to unrecorded SpO _2_ data. Our analysis, presented in Table 3, suggests that oxygen saturation was MNAR as an unrecorded SpO _2_ was an independent mortality risk factor.

**Table 3.** Evaluation if SpO _2_ recordings are Missing Not at Random [MNAR] (N=13,413 Deaths= 373).

Variable	Adjusted Odds Ratio	95% Confidence Interval
SpO _2_ < 90%	4.0	3.0,5.3
Unrecorded SpO _2_	4.9	3.8,6.3
-3<WAZ≤-2	2.8	2.0,3.8
WAZ≤-3	2.4	1.7,3.3
Female	1.3	1.0,1.6
Chest indrawing	1.0	0.6,1.6
Wheezing	0.7	0.5,1.0
WHO Danger Signs ^a^	4.3	3.3,5.6
Under 12 months old	1.2	1.0,1.5

SpO _2_: peripheral oxyhemoglobin saturation, WAZ: WHO weight-for-age z-score; ^a^Danger signs include unable to drink/feed, convulsions, sleepy/lethargic, vomiting everything, stridor

## Discussion

We explored the impact of applying the 2014 WHO IMCI guidelines, using a large existing dataset of children admitted to hospital with clinically defined pneumonia. If the 2014 WHO IMCI guidelines are implemented without compulsory pulse oximetry measurement of children evaluated for pneumonia in Malawian outpatient settings, some children that could benefit from inpatient oxygen treatment will likely be missed. This could result in increased child pneumonia deaths. A direct comparison of non-severe, outpatient pneumonia CFRs showed a two-fold higher CFR when using the 2014 rather than the 2005 guidelines without pulse oximetry, 1.5% vs 0.8% (P=0.052). However, implementation of the 2014 IMCI guidelines with compulsory pulse oximetry decreased non-severe pneumonia CFR by 50%, to 0.7% for this group.

We found that the inability to obtain a pulse oximetry reading was MNAR, suggestive as an independent mortality risk factor. We found a similar finding amongst hospitalized Malawian infants under 2 months old ^14^. In a separate study evaluating pulse oximetry use in outpatient settings, we found that 1 out of the 32 cases that were not clinically eligible for referral and had an unrecorded pulse oximetry level died within 30 days ^9^. It is challenging to interpret this finding because although we witnessed study staff struggle with obtaining a pulse oximetry reading in some children, it is plausible that these data may be missing not at random but for other reasons. For instance, clinicians may not have paused to measure a pulse oximetry reading in children who presented to care when obviously critically ill. Biologically, the inability to measure pulse oximetry could reflect poor peripheral perfusion, which occurs in high systemic vascular resistance states such as shock. There are ongoing studies evaluating if a peripheral perfusion index, derived from the plethysmography signal of pulse oximetry, could be used as an early marker of microcirculatory dysfunction during states such as sepsis and compensated hypovolemic shock before changes in mean arterial blood pressure, which is currently used to determine macrovascular collapse ^17– 19^.

In contrast with Agweyu’s findings from Kenya, we were able to explore the potential implications of the 2014 guidelines in the context of pulse oximetry. Our study has similar findings, using retrospective data from hospitalized cases, in that there was an unacceptably high CFR amongst children diagnosed with non-severe pneumonia using the 2014 guidelines without pulse oximetry. There are some notable differences between the study populations, with the Kenyan study reporting a higher overall CFR (5.2% vs 2.8%), a higher CFR in children classified as non-severe pneumonia (2.7% vs 1.5%), and fewer cases from malaria-endemic regions (33% vs 100%) ^2^. These are important distinctions as the case fatality difference could be attributed to a variety of factors, including an improved survival benefit in Malawi with inpatient use of pulse oximetry and subsequent oxygen treatment. While this is conjecture, the approximate 50% reduction in mortality between the settings is in line with findings from Nigeria, showing a similar reduction in inpatient pneumonia mortality following pulse oximetry introduction ^20^. Another explanation could be the difference in malaria prevalence amongst these populations, as the IMCI guidelines for pneumonia diagnosis overlap with the clinical diagnosis of malaria ^21^. Given that they did not have pulse oximetry data and our dataset did not include information about dehydration or pallor, direct comparisons of independent risk factors for pneumonia-associated mortality between the two studies is problematic. Though we do report mortality risk factors in this study, the intention was solely to explore if pulse oximetry was MNAR. We have previously published our findings, using this same dataset, on pneumonia related mortality risk factors and developed a clinical prediction rule, the Respiratory Index of Severity in Children – Malawi (RISC-Malawi), though excluding patients without a recorded pulse oximetry reading ^22^. An important finding was that girls had a higher adjusted odds for in-hospital pneumonia related mortality.

Notably the Kenyan and our studies report fewer girls were hospitalized with pneumonia, though female sex was an independent mortality risk factor. We observed the same pattern when examining pneumonia related mortality risk factors in Malawian infants under two months old, and this is consistent with a large metanalysis of acute lower respiratory illness mortality risk factors ^14, 23^. However, in our previous Malawian studies, utilizing data collected concurrently with this study, in outpatient settings (community health workers and health centres), we found that similar proportions of boys and girls, aged 0–59 months old, sought evaluation for pneumonia ^9, 14^. Our incidental findings that an equal proportion of girls and boys presented for outpatient pneumonia care, fewer girls were hospitalized, yet being a girl was an independent pneumonia-related mortality risk factor cannot go ignored. These findings are consistent with those of a retrospective qualitative study assessing care seeking patterns in the same region of Malawi (Mchinji) that suggested that girls presented to the hospital more often than boys but were admitted less often ^8^. Differences in the type of care girls receive and their outcomes must be examined.

Pulse oximetry implementation, while crucial to health systems strengthening, is not without challenges. First, more work must be done to identify context appropriate SpO _2_ thresholds at which to refer patients for hospital evaluation, hospitalize patients, and initiate or wean supplemental oxygen. Previous findings suggest that moderate hypoxemia of SpO _2_ 90–92% is a mortality risk factor in Malawian children under 5 with acute lower respiratory infections ^22, 24^. One hypothesis for this finding is that pulse oximeters may be inaccurate in children with dark skin tones. A research report from the United States suggested that pulse oximetry may over-estimate SpO _2_ in adults with dark skin, potentially leading to missed hypoxemic cases and further exacerbating racial health disparities ^25, 26^. While this study had some methodological limitations such as using paired pulse oximetry and arterial blood gas readings that were time-delayed by up to 10 minutes and only adjusting for poor peripheral perfusion in one single center cohort, this concept warrants investigation in children. While there are clinical trials in low- and high-income countries exploring the optimum SpO _2_ threshold for initiation of supplemental oxygen, they have prompted debate on the ethics of such studies and, at the time of writing, have not reported findings ^27, 28^.

Second, pulse oximetry programs must be implemented with a clearly defined clinician mentoring and longitudinal implementation plan. Even when available in health facilities, barriers exist to pulse oximeter utilization ^29, 30^. Enoch *et al.* conducted a comprehensive, multi-site, mixed methods study in Kenya to assess pulse oximetry use and limitations to its uptake by different types of healthcare workers ^31^. They found an interdependence of pulse oximetry usage and oxygen therapy availability. This suggests that, in order to ensure usage, pulse oximetry must be implemented in concert with supplemental oxygen and other types of respiratory support, rather than as a separate health system intervention.

Cost savings from reduced hospitalizations at both the household and health system levels, compounded with the reduced clinical burden on already limited hospital staff, may have significant spillover effects that we cannot capture in this analysis. It is plausible that, in certain settings, pulse oximetry implementation may in effect pay for itself by reducing downstream costs. As health systems move towards universal integration of pulse oximetry, these potential savings should be accounted for in order to make accurate comparisons with other high priority interventions.

The main limitation of this analysis is that we extrapolate potential outpatient outcomes from hospital inpatient data. We may be underestimating, to varying degrees, the true CFR of these groups. Hospitalized patients may have received more effective interventions, such as intravenous antibiotics, that they would not have received if discharged home. Alternatively, hospitalization may place these children at risk of contracting hospital-acquired infections and other communicable diseases. We found that pulse oximetry recordings were MNAR. As we have previously reported, it is unclear if these data are missing because patients were critically ill and clinicians were unable to obtain an appropriate pulse oximeter read due to poor perfusion or if they simply did not record the measurement ^12^. Our anecdotal experience suggests the former scenario is not uncommon. In real world clinical settings, not all children with severe disease present with the limited criteria in the IMCI guidelines. Clinician judgement of illness severity was not recorded in this data set, so we are unable to determine how many cases classified as non-severe would have been hospitalized regardless of the guideline recommendation.

These data strongly support compulsory pulse oximetry use if Malawi is to fully adopt the 2014 IMCI pneumonia guidelines in outpatient facilities. Our findings suggest that the implementation of the 2014 IMCI guidelines would likely result in a marked decrease in under-five pneumonia related hospitalizations. We would recommend that children under 5 years old with chest indrawing in whom pulse oximetry is unable to be obtained, still warrant referral for further evaluation. It is imperative for country level ministries of health in other LMICs to assess the potential effects of the 2014 policy changes in their own settings before widespread implementation. In Malawi, implementation of the 2014 WHO IMCI guidelines, without standard pulse oximetry measurement in children evaluated for pneumonia in outpatient settings may result in missed hospital referral opportunities of high-risk cases.

## Data Availability

The data is owned by the Malawi Ministry of Health. As such, and given that the data is not de-identified, readers/reviewers must apply for access to the data. Enquiries for data access should be addressed to Mr. Norman Lufesi ( nlufesi@gmail.com) the Acting Chief of Emergency and Critical Care Services of the Malawi Ministry of Health who will facilitate access. Data access will be granted after approval by the Malawi Ministry of Health and the Malawi National Health Science Research Committee. Figshare: STROBE checklist for ‘Implication of the 2014 World Health Organization Integrated Management of Childhood Illness Pneumonia Guidelines with and without pulse oximetry use in Malawi: A retrospective cohort study’. https://doi.org/10.6084/m9.figshare.21346797 ^32^. Data are available under the terms of the Creative Commons Zero "No rights reserved" data waiver (CC0 1.0 Public domain dedication).
