# Supplementary material for: Implication of the 2014 World Health Organization Integrated Management of Childhood Illness Pneumonia Guidelines with and without pulse oximetry use in Malawi: A retrospective cohort study
Source: Gates Open Res. 2023 Nov 8;7:71. Originally published 2023 May 15. [Version 2] doi: 10.12688/gatesopenres.13963.2 (PMC10651692; doi:10.12688/gatesopenres.13963.2)
Supplement: Peer review history

# Reviewer report: Reviewer response for version 2

**DOI:** 10.21956/gatesopenres.16452.r35506

**Published:** 2023-11-15

**Authors:** Kimberly Baltzell

**Affiliations:** 1 Department of Family Health Care Nursing, Institute of Global Health Sciences, University of California San Francisco, San Francisco, CA, USA

I feel the revisions were adequate and I approve the new version.

Is the work clearly and accurately presented and does it cite the current literature?

Yes

If applicable, is the statistical analysis and its interpretation appropriate?

I cannot comment. A qualified statistician is required.

Are all the source data underlying the results available to ensure full reproducibility?

Yes

Is the study design appropriate and is the work technically sound?

Yes

Are the conclusions drawn adequately supported by the results?

Yes

Are sufficient details of methods and analysis provided to allow replication by others?

Partly

Reviewer Expertise:

NA

I confirm that I have read this submission and believe that I have an appropriate level of expertise to confirm that it is of an acceptable scientific standard.

# Reviewer report: Reviewer response for version 1

**DOI:** 10.21956/gatesopenres.15257.r33639

**Published:** 2023-07-19

**Authors:** Amy Gray

**Affiliations:** 1 Department of Paediatrics, The Royal Children's Hospital Melbourne, The University of Melbourne, Victoria, Australia

Thank you for the opportunity to review this paper which addresses an important topic.

Background:

 I thought the background outlined succinctly the key challenges and changes related to pneumonia classification and clinician management decisions. Well done.

Methods:

 I presume the data was collected from this set available from 2012-14 as it predates the change in IMNCI guidelines. It would be good to be explicit about this intention if this is true.

Results:

 These are well described with appropriate supporting tables. 

Discussion:

 Good summary of findings in first paragraph and comparison with other literature in subsequent paragraphs. With good reference to practical considerations. 

 Overall I throroughly enjoyed reading this publication, its findings and discussion. Some minor corrections:

Study design:

 - "provided intermittently."

Discussion:

- Paragraph 1: remove "from" 1.5% vs 0.8%
- Paragraph 4: Notably (remove "both") the Kenyan and our studies...as there are more than 2? Or is it only referring to two?
- Paragraph 4: concurrently "with" this study...
- Paragraph 6:..."respiratory" support.

Is the work clearly and accurately presented and does it cite the current literature?

Yes

If applicable, is the statistical analysis and its interpretation appropriate?

Yes

Are all the source data underlying the results available to ensure full reproducibility?

Yes

Is the study design appropriate and is the work technically sound?

Yes

Are the conclusions drawn adequately supported by the results?

Yes

Are sufficient details of methods and analysis provided to allow replication by others?

Yes

Reviewer Expertise:

Paediatrics, quality of care for children including oxygen systems, health professional education.

I confirm that I have read this submission and believe that I have an appropriate level of expertise to confirm that it is of an acceptable scientific standard.

# Reviewer report: Reviewer response for version 1

**DOI:** 10.21956/gatesopenres.15257.r33354

**Published:** 2023-06-20

**Authors:** Kimberly Baltzell

**Affiliations:** 1 Department of Family Health Care Nursing, Institute of Global Health Sciences, University of California San Francisco, San Francisco, CA, USA

The topic is important and needs study such as this one.

**Background:** the background was compelling and complete and makes the case for this sort of study. It was not clear to me why the study was published so long after data was collected.

**Study Design: **While the study did not include patients <2 months of age, IMCI was updated in 2019 to include significant changes for managing such patients. This would be worth mentioning. To note, there were several typos in this section requiring a careful proof-read.

**Methods: **The authors mention the team was supervised to ensure activities were carried out properly. However, there was no mention of who was doing the supervising, if supervision was constant, e.g. including night shift. In addition, it would be helpful to know what sort of remediation occurred if something was amiss. I would appreciate knowing more about the pulse ox that was used, the accuracy and use in other similar populations. For the population of interest, were these only patients that were hospitalized directly or hospitalized after referral from a health facility. If they were two different groups, it would be curious to know if those who presented directly to the hospital had better outcomes than those who were admitted only after referral from a health facility if this information is available.

**Results:** no comments.

**Discussion:** It was important to add information about skin pigment and its impact on pulse ox readings. As mentioned earlier, perhaps more information on the pulse ox used and the justification for its use at that point in time. Also, was there follow up of the children who were not referred to see if there were poor outcomes in that group at a later time? In other words, did the patients who were not referred were assumed to have recovered?

**General:** Thank you for a very interesting paper. As mentioned above, I am curious why there was such a long time since the collecting of the data and the publication, especially given the changes in IMCI since these data were collected. And if IMCI revisions in 2014 led to worse outcomes in some groups, it is important to share the information sooner rather than later.

Is the work clearly and accurately presented and does it cite the current literature?

Yes

If applicable, is the statistical analysis and its interpretation appropriate?

I cannot comment. A qualified statistician is required.

Are all the source data underlying the results available to ensure full reproducibility?

Yes

Is the study design appropriate and is the work technically sound?

Yes

Are the conclusions drawn adequately supported by the results?

Yes

Are sufficient details of methods and analysis provided to allow replication by others?

Partly

Reviewer Expertise:

NA

I confirm that I have read this submission and believe that I have an appropriate level of expertise to confirm that it is of an acceptable scientific standard, however I have significant reservations, as outlined above.
